# Vivisection

**Published:** 1866-12

**Authors:** 


### SELECTED ARTICLES.

*Vivisection.*—The practice of vivisection, of conducting physiological experiments on living animals, of laying bare the organs within the body and demonstrating to the eye the vital processes and changes which otherwise must be left very much to conjecture and theory, is one which, considered purely in itself, naturally excites the strongest repugnance in every mind which lays the slightest claims to humanity. The sight of the suffering thus inflicted on the lower animals cannot but cause a shudder, and it requires a conscientious purpose on the part of the operator and an intelligent conviction on the part of the spectator that an investigation is being made the object of which is the physical welfare of a higher race, to justify it or to make it even tolerable. Doubtless there have been in times past, too much reckless experimentation of this kind, too frequent repetition of the same experiment, too great readiness for mere purposes of display to exhibit what has already been sufficiently demonstrated, and too little consideration for the sufferings thus unnecessarily produced. As we have said, it requires a full appreciation of the importance of the object sought for to make such investigations tolerable. It is not, therefore, a difficult thing to so describe them, particularly to persons not professionally interested, as to make them to their minds the very consummation of everything barbarous and cruel. This view may, nevertheless, be as unwise and unjust as the criminations of those who stigmatize the operations of the noble science of surgery as nothing better than butchery.

Influenced by such feelings, it is not surprising that movements should have been made at different times on the part of benevolent persons to check an undue tendency to the repetition of such experiments, or even to prevent them altogether by legal enactment. In this country, until quite recently, very little of this method of pursuing physiological study has been done. Professor Dalton, of the College of Physicians and Surgeons in New York, has probably made more researches in this way than any other American physiologist. The value of the results which he has obtained has been universally acknowledged by the medical profession ; and as it is every day becoming more and more evident that the true practice of medicine must be based on the most accurate physiological knowledge, the value of the information gained in this way is becoming more and more demonstrated. Prof. Dalton's vivisections, however, have attracted the attention of the New York Society for the Prevention of Cruelty to Animals, and its President,

Mr. Henry Bergh, has felt it his duty to address a letter of inquiry on the subject to Dr. Delafield, President of the College of Physicians and Surgeons, suggesting that if vivisections could not be altogether dispensed with, they might at least be deprived of their suffering by the use of anæsthetics. Dr. Delafield referred this letter to Dr. Dalton, who replied that he was in the habit of using anæsthetics in all instances which it was possible without defeating the object of the experiment. This statement was not specific enough to satisfy Mr. Bergh, who wrote another letter, asking information concerning the exceptional cases; which Dr. Dalton explained by stating that they were cases where the object was "to ascertain the presence or absence of sensibility in particular parts."

It seems that these statements were still unsatisfactory, and Mr. Bergh accordingly addressed a long letter to Dr. Delafield, setting forth in the strongest terms his abhorrence of all such practices, giving a detailed description of some of Magendie's experiments, presenting the opinions of prominent members of the medical profession in Europe against them, and concluding with a protest "in the name of Heaven, public morality and of this Society against these fearful cruelties, inflicted on dumb, unresisting creatures, confided to the merciful protection of mankind, without the employment of anæsthetics." The letter is published in full in the N. Y. *Evening Post* of the 7th inst.

Making all due allowance for the humane feeling which has actuated Mr. Bergh, we feel that in this matter he has gone too far. It is impossible for an unprofessional mind fully to appreciate the considerations which justify to a physiologist what considered by itself merely is but a painful spectacle of suffering. We feel that he should have trusted to the humanity of Dr. Dalton to pursue his researches in the true spirit of scientific inquiry, the sole object of which is the ultimate diminution of human suffering. Least of all was he justified, in our opinion, in printing his letter in the daily press, to excite the universal horror and indignation of sensitive minds. As well might he have opened the doors of a dissecting room to the public for the purpose of producing a prejudice against the indispensable study of practical anatomy. Dr. Dalton replied to Mr. Bergh's letter as follows, and we leave his justification in his own hands; we find it in the New York *Evening Post* of the 8th inst.

College of Physicians and Surgeons,
New York, November 8, 1866.

*Edward Delafield, M.D., President of the College of Physicians and Surgeons:*

Dear Sir,—I have received your letter of this date, enclosing a third communication from Mr. Henry Bergh, President of the Society for the Prevention of Cruelty to Animals, dated November 1, in which he enters at length into the question of the propriety of vivisections, and in which he protests, "in the name of Heaven, public morality, and of this Society, against these fearful cruelties, inflicted on dumb unresisting creatures, confided to the merciful protection of mankind, without the employment of anæsthetics." His letter, I see, is also published in the New York *Evening Post* of yesterday.

Without replying to the extravagant epithets contained in Mr. Bergh's letter, which are of no consequence in themselves, and which will have no weight with those who desire to be correctly informed as to the facts in the case, I desire to mention to you a few considerations which show that the practice of experimenting upon animals for physiological and medical purposes, which is held up to so much animadversion, is an eminently proper one; and so far from deserving reprobation, is entitled to respect as a valuable and legitimate means of benefiting mankind.

First of all, its sole and ultimate object is the relief of human suffering and the cure of human diseases. It is the best and most valuable means by which our knowledge of physiology is increased; and upon physiology the cultivation and improvement of the whole medical art depend. All intimations that it has any other motive than this—such as display wantonness, or the indulgence of a reckless cruelty, are false, and do not represent its true character. The knowledge which is obtained by it enables us to understand the natural functions, without which the study of medicine in all its branches would be retarded, and indirectly but certainly the success of medical practice diminished. In point of fact, nothwithstanding all unfounded assertions to the contrary, the greatest discoveries in physiology have, in past times, being directly due to its employment; and while we owe to it a large proportion of the useful knowledge which we now possess, it would be the greatest misfortune for medicine and the welfare of mankind, if it were abandoned or neglected in the future.

Secondly, it is not a cruel practice, but may be and is, in the great majority of instances, conducted in a perfectly humane and unobjectionable manner. It can never be the objecte of th physiologist to inflict unnecessary suffering; but on the contrary, it is desirable both for the sake of humanity and for the attainment of his object to avoid doing so. This is greatly facilitated by the use of anæsthetics. Since the anæsthetic properties of ether and chloroform have been known to us, their employment has been fully as useful to the physiologist as to the surgeon, and they enable us to do the requisite préliminary operations without the animal experiencing any sense of suffering, and even without his conciousness.

As you are aware, it has been my constant practice to employ ether for this purpose, in the College of Physicians and Surgeons, whenever the operation to be performed was calculated to inflict pain upon the animal. This can always be done, except in a very small class of cases, where the aim of the experiment being to ascertain the existence of sensibility in a particular part, the unconsciousness produced by ether would defeat the object of the operation.

This is especially true in certain experiments of Magendie, performed in 1822, on the roots of the spinal nerves, to which I referred in a former letter, and by which the seat of sensibility in these parts was ascertained. But these experiments are so rare that I have not only never had occasion to do them myself, but do not recollect ever to have seen them performed. Yet I know that they have been done in former times, and have been productive of the most valuable and lasting results; and when requisite for other objects similar experiments might again become necessary and proper, and even here the amount of suffering inflicted is very much less than it is often represented to be. It can only be necessary to obtain an indication of sensibility, and the object of the experiment is accomplished. The practice of performing surgical operations on living animals by students or practitioners as a means of acquiring dexterity, so far as I am aware, is unknown in this country.

I allude to these considerations because I think they are important to the cause of medical education, in which we, as well as the whole community, are interested. In the College of Physicians and Surgeons over which you preside, it has always been believed that the fullest instruction in physiology, as well as in the other elementary branches, is essential to the due preparation of intelligent and successful physicians. The more complete and efficient this instruction is made, the more competent will be the practitioners who every year are sent out to join the ranks of the medical profession. This instruction is made as complete as possible by ocular demonstrations of many important facts, but this is always done, as I believe, in a reasonable and proper way. It is not the case that "revolting barbarities are repeated at lectures for the mere gratification of juvenile curiosity." It is unnecessary to say to those who are familiar with these lectures that no such barbarities are to be seen there; but it may be desirable for the information of those who are unacquainted with the subject, and might otherwise acquire an unfounded prejudice against it. I cannot, therefore, pass over without reply the communication of Mr. Bergh, which is calculated to place in so false a light one of our most valuable means of improvement in medicine.

Yours, very respectfully, J. C. DALTON, M.D.., *Prof. of Physiology.*

—*Boston Medical and Surgical Journal*, Nov. 29th, 1866.

